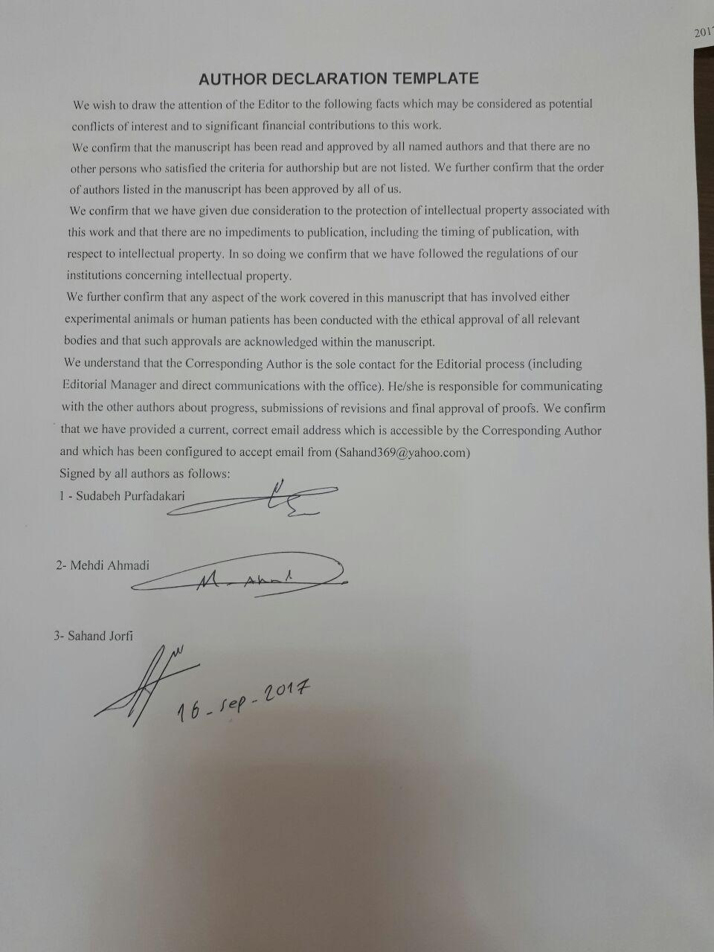# Experimental data on adsorption of Cr(VI) from aqueous solution using nanosized cellulose fibers obtained from rice husk

**DOI:** 10.1016/j.dib.2017.10.043

**Published:** 2017-10-31

**Authors:** Sudabeh Pourfadakari, Sahand Jorfi, Mehdi Ahmadi, Afshin Takdastan

**Affiliations:** a Department of Environmental Health Engineering, School of Health, Ahvaz Jundishapur University of Medical Sciences, Ahvaz, Iran; b Environmental Technologies Research Center, Ahvaz Jundishapur University of Medical Sciences, Ahvaz, Iran

**Keywords:** Rice husk, Nano-sized cellulose, Cr(VI), Adsorption, Water pollution

## Abstract

The aim of this study was to evaluate the efficiency of nano-sized cellulose obtained from rice husk for Cr(VI) adsorption. The effect of operational parameters including initial pH (3–10), contact time (0–120 min), adsorbent dosage (0.2–1.5 g/L), and initial Cr(VI) concentration (5–50 mg/L) were investigated according to one factor at time method. The results showed, in pH=6, contact time=100 min, adsorbent dose=1.5 g/L and 30 mg/L initial chromium concentration, the adsorption efficiency reached to 92.99%. Also Langmuir isotherm with (R^2^=0.998 at 303 °K) and pseudo-first-order kinetic model (R^2^=0.993) were the best models for describing the Cr(VI) adsorption reactions. The negative values of ΔG∘ and positive value of ΔH∘ showed that, the Cr(VI) adsorption on NCFs was endothermic and spontaneously process. Therefore, it can be concluded that the application this method is recommended for removing Cr(VI) from aqueous solutions.

**Specifications Table**

**Table:** 

Subject area	*Water pollution*
More specific subject area	*Water and wastewater treatment*
Type of data	*Table and Figure*
How data was acquired	*Experiments were performed according to a designed procedure and output of analytical test were processes in order to perform an analysis of adsorption process.*
Data format	*Processed*
Experimental factors	*Studied variables included pH, contact time, adsorbent dosage, and Cr(VI) concentration which were investigated for Cr(VI) adsorption*
Experimental features	*Adsorption of Cr(VI) in a synthetic sample was studied using synthetized nano- cellulose*
Data source location	*Ahvaz city, Khuzestan province, Iran*
Data accessibility	*Data are available in article*

**Value of the data**

• Data are benefit for determination of the isotherm, kinetic, and thermodynamic data and also for predicting and modeling the adsorption capacity and mechanism of chromium (VI) removal by the adsorbent will applicated.
• A simple method used for preparation of nano- cellulose fibers from rice husk.
• The dataset will be useful for Cr(VI) ion removal from water and wastewater.

## 1 Data

**Fig. 1:**
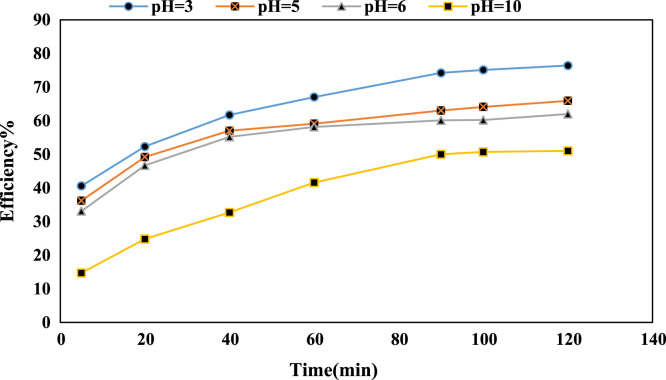
Effect of pH on Cr(VI) removal efficiency (NCFs dosage: 0.5 g/L, and Cr(VI) concentration: 30 mg/L).

**Fig. 2:**
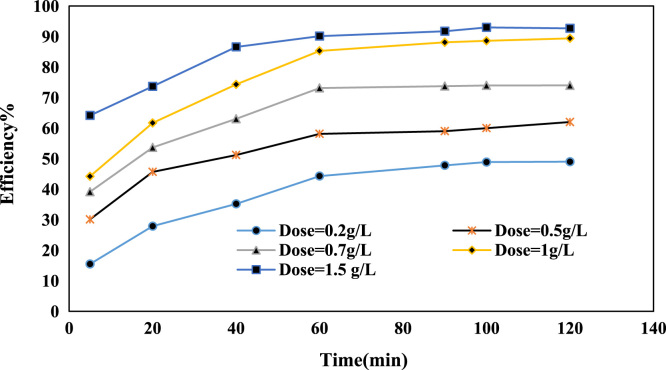
Effect of NCFs dosage on Cr(VI) ion removal at (pH: 6, Cr(VI) concentration: 30 mg/L).

**Fig. 3:**
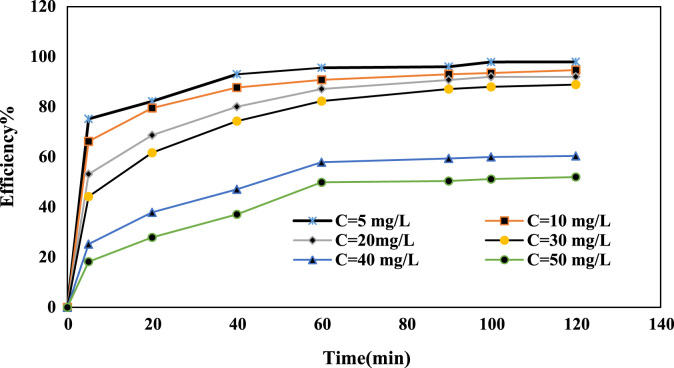
Effect of the initial Cr(VI) concentration on Cr(VI) ion removal (pH: 6, NCFs dosage: 1 g/L).

**Fig. 4:**
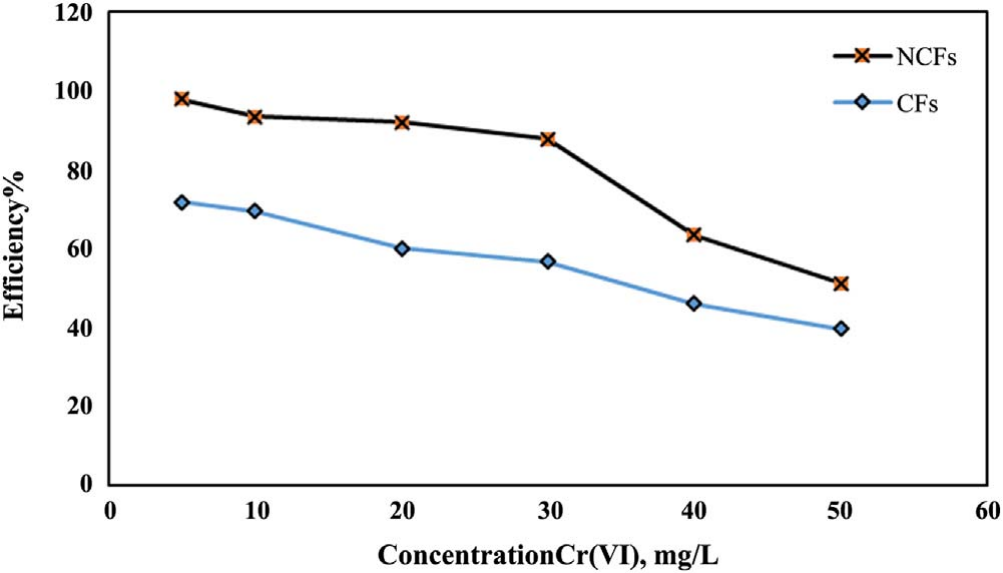
Compare the effect of cellulose fibers (CFs) and NCFs on Cr(VI) ion removal (pH: 6, adsorbent dosage: 1 g/L, at contact time 100 min.

**Fig. 5:**
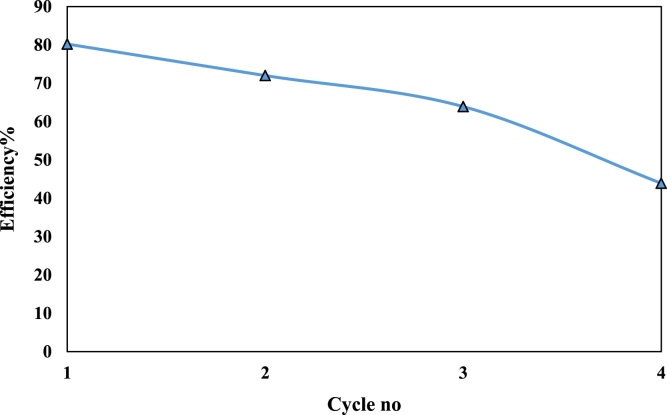
Desorption of nano cellulose fibers using solution 0.5 M of HNO_3_ for Cr(VI) ion removal (30 mg/L).

**Fig. 6:**
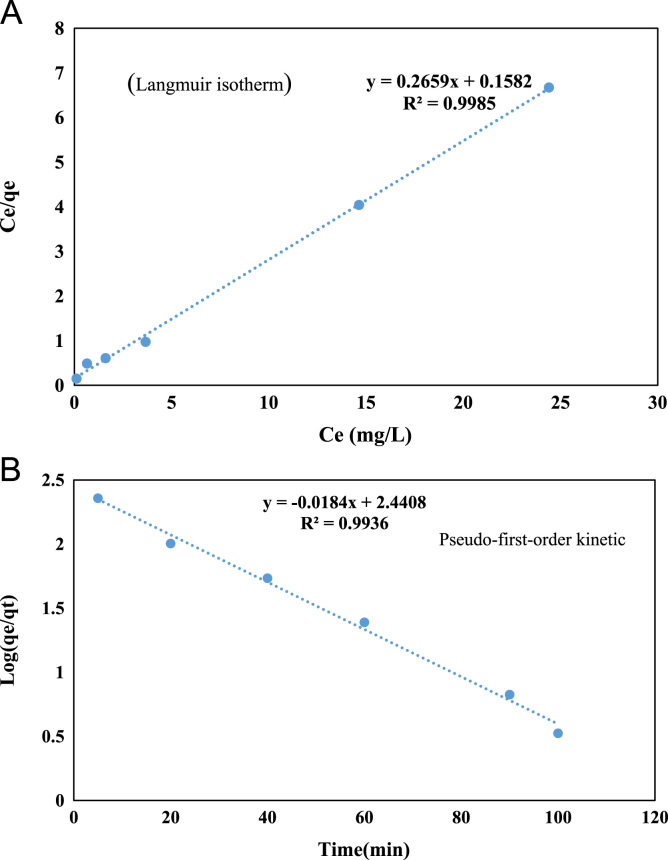
Modeling **A)** Langmuir Isotherm and **B)** Pseudo first-order Kinetic Model for Cr(VI) adsorption using NCFs at (pH: 6, NCFs dosage: 1 g/L, Cr(VI) (Concentration: 5– 50 mg/L).

**Fig. 7:**
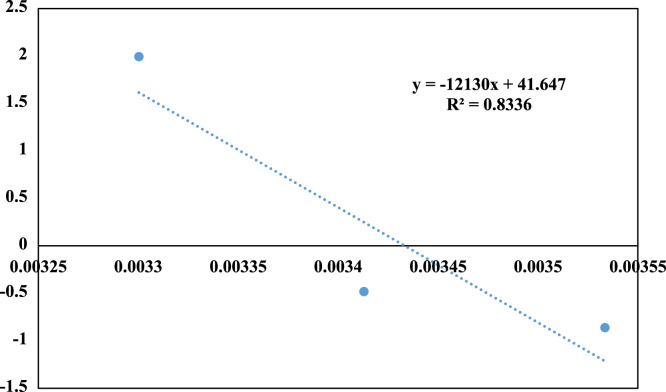
Thermodynamic profile for Cr(VI) adsorption onto NCFs. Temperature range = 283–303 °K, Cr(VI) concentration = 30 mg/L, pH = 6.0, contact time = 100 min, and adsorbent dosage = 1 g/L.

This data set contains 7 Tables and 7 Figure. Fig. 1, Fig. 2, Fig. 3, Fig. 4, Fig. 5 shows the effect of different parameters on the removal of chromium with nano- cellulose fibers. Also, Tables 1 and 2 shows isotherm and kinetic equations and the coefficient of correlation this equations is presented in Table 3, Table 4. Fig. 6, Fig. 7 shows adsorption isotherm and kinetic curve and regressions of vant Hoff plot for thermodynamic parameters.

**Table 1:** Characteristics of the isotherms.

Type of isotherm	Equation	Linear form	
Freundlich	$q_{e}=K_{f}{C_{e}}^{\frac{1}{n}}$	$\log\;q_{e}=\;\log\;K_{f}+\left(\frac{1}{n}\right)\log\;C_{e}$	(4)
Langmuir	$q_{e}=\frac{Q_{m}K_{L}C_{e}}{1+K_{L}C_{e}}$	$\frac{C_{e}}{q_{e}}=\left(\frac{1}{K_{L}Q_{m}}\right)+\left(\frac{1}{Q_{m}}\right)C_{e}$	(5)
	
Temkin	$q_{e}=B1Ln(K_{T}C_{e})$	$q_{e}=B_{1}\;\ln\;K_{T}+B_{1}\;\ln\;Ce$	(6)
D-R		$Lnq_{e}=\;\ln\;q_{m}-\beta \varepsilon 2\;$	(7)

**Table 2:** Kinetic equations and linear forms used in this study.

Kinetics of	equation	linear form	
Pseudo-first-order	$\frac{dq_{t}}{dt}=k_{1}(q_{e}-q_{t})$	$\log\left(q_{e}-q_{t}\right)=\;\log\left(q_{e}\right)-\frac{k_{1}}{2.303}t$	**(8**)
Pseudo-second-order	$\frac{dq_{t}}{dt}=k_{2}{\left(q_{e}-q_{t}\right)}^{2}$	$\frac{t}{q_{t}}=\left(\frac{1}{k_{2}q_{e}^{2}}\right)+\left(\frac{1}{q_{e}}\right)t$	(9)

**Table 3:** The results of calculations of the adsorption isotherm.

Langmuir			Freundlich				Dubinin RadushTkevich (D-R)				Temkin		

q_max_(mg/g)	K (L/mg)	R^2^	n	k_F_ (mg/g)	R^2^	β		E	Qm	R^2^	bt	K_T_	R^2^
3.76	0.042	0.998	3.16	1.71	0.87	4*10^-8^		3.2	3.035	0.80	1786	7.71	0.857

**Table 4:** The results of studying the kinetics.

Type of kinetic model	Parameter	Value
		0.042
	q_e_cal	275.42
	R^2^	0.993
Pseudo-second-order	k_2_	0.0002
	q_e_cal	500
	R^2^	0.931

## 2 Experimental

### 2.1 Materials

Rice husk used in this study was prepared from Northern of Iran. Sodium chlorite (NaClO_2_), acetic acid glacial (CH_3_COOH), potassium hydroxide (KOH), sulfuric acid (H_2_SO_4_) and the other chemicals used in this study were prepared from Merck Germany and used without additional treatment.

### 2.2 Experimental procedure

The adsorption experiments were carried out in laboratory scale on synthetic wastewater, inside Erlenmeyer flasks with volume of 200 mL. Besides, HCl and NaOH 1 N were used in order to adjust the pH level at the beginning of each experiment. Mixing was performed using a shaker incubator with 150 rpm at the temperature range of (283–303 °K). Effect of operational parameters including as pH (3–10), reaction time (0–120 min), initial Cr(VI) concentration (5–50 mg/L) and adsorbent dosage (0.2–1 g/L) were assessed. To determine the residual concentration of Cr(VI), samples were centrifuged at 3000 rpm for 10 min. Thereafter the supernatant was used for analysis residual Cr(VI) concentration by flame atomic absorption spectroscopy (FAAS) (Model AAS vario6 Jena, Germany). The Cr(VI) concentration was determined according to standard methods for examination of water and wastewater [1].

In addition, the adsorption capacity (mg/g) and adsorption efficiency (%) were obtained using Eqs. (1), (2) [2]:

(1) qe=(C0−Ct)×V/M

(2) Re(%)=(C0−Ct)/C0×100

Where q_e_, is the amount of Cr(VI) adsorbed (mg/g), C_0_ and C_t_ are initial and final Cr(VI) concentrations, V is the volume (L), and M, is the adsorbent dosage (g).

### 2.3 Preparation of nano-sized cellulose fibers (NCFs)

NCFs were prepared according to the technique given by Lu et al. [3], with some modifications. Briefly, at the first, to remove dirt and soluble substances, rice husk were washed with distilled water four times, and dried overnight in oven at temperature of 313 °K. Then rice husk crushed to smaller pieces of (5–10 mm) through a grinder and passed of 60-mesh screen. Afterwards, 30 g of product was soaked in proportion 2:1(v/v) toluene/ethanol (450 mL) mixture for 20 h to remove impurities such as oil and wax, then dried in at 328 °K for 24 h. the dewaxed fibers were immersed in sodium chlorite solution (pH= 4) for 1 h at 323 °K to remove lignin and then washed with distilled water. Hemicellulose and pectin were treated with 600 mL solution of 5% KOH for 24 h and dried at temperature 363 °K for 2 h and then washed with distilled water. Cellulose isolated was hydrolyzed using acid hydrolyzed (40 ml DI water + 20 ml HCl 12.1 N and 40 ml H_2_SO_4_ 36 N) for 3 h at 343 °K for to obtain soft wood pulp and then washed with distilled water. Finally these fibers were sonicated (Hielsccher: UP 400S, Germany), operating at a fixed frequency of 50 KHZ at 353 °K for 3 h, dried and subjected for microscopic analysis (Table 5, Table 6, Table 7).

**Table 5:** Thermodynamic parameters at different temperatures.

T(°K)	ΔG∘_(KJ/mol)_	ΔS∘_( KJ/mol)_	ΔH∘_(KJ/mol)_
283	-0.198	0.346	100.84
293	-0.202		
303	-0.205

**Table 6:** Adsorption of Cr(VI) by different lignocellulose wastes.

**Adsorbent material**	**Optimum Conditions**	**Q**_**max**_**(mg/g)**	**Removal (%)**	**References**
Rice Husk Carbon	Time=240 min, pH=2	38.1 mg/g	93–94	[14]
Bagasse fly ash	Time=40 min, pH=5	1.8	96–98	[15]
Hazelnut shell	pH<3	17.7	97.8	[16]
Oat biomass	Time=120 min, pH=2	10.92	32	[17]
Raw rice bran	Time=60 min, pH=4	–	40–50	[18]
Coconut shell fibers	Time=180 min, pH=6	–	>85	[19]
Tea factory waste	Time=60 min, pH=2	54.65	37–99	[20]
tamarind seeds	pH=2–3 Time=60 min	29.7	98	[21]
maize bran	pH=2 Time=180 min	312.59	>80	[22]

**Table 7:** Notation used in the kinetic models and the adsorption isotherms.

**Nomenclature**	
K_L_	Langmuir isotherm constants (L/mg)
K_f_	Freundlich isotherm constants (L/g)
n	Adsorption intensity
q_t_	Adsorbed metal concentration at time t (mg/g)
C_e_	Equilibrium concentration in solution (mg/L)
q_e_	Equilibrium adsorbent concentration on adsorbent (mg/g)
q_e_cal	Calculated values of q_e_ (mg/g)
Q_m_	Maximum monolayer capacity (mg/g)
R^2^	Correlation coefficients
K_1_	Pseudo first-order rate constant (1/min)
K_2_	Pseudo second-order rate constant (g/mg min)
K_dif_	Intraparticle diffusion rate constant (mg/g min^0.5^)
β	Activity coefficient constant(mol^2^/j^2^)
Ɛ	Polanyi potential

### 2.4 Desorption study

In order to predict reusability of NCFs, four adsorption–desorption cycles were considered. The adsorption was performed using an initial Cr(VI) concentration of 30 mg/L. At the first, metal loaded NCFs obtained from experimental was poured into the Laboratory jar that was contain 50 ml of HNO_3_ 0.5 M and rocked for 20–100 min. Then, the sample was centrifuged and using Whatman 42 filter paper to remove any excess of Cr(VI) in the surface of the NCFs was filtered , and regenerated adsorbent for removal Cr(VI) was used [4]. Desorption ratio (DR %) was calculated through Eq. (3), [5].

(3) DR%=Cdes/Cad×100

where: C_des_ (mg/L) is the amount of desorbed metal ion, and C_ad_ is amount of adsorbed metal ion in solution.

### 2.5 Adsorption isotherms

In the current study, the experimental data of adsorption equilibrium were investigated using Langmuir, Ferundlich, Temkin and Dubinin–Radushkevich (D-R) isotherm models. The study of isotherm models were carried out in pH of 6, adsorbent dosage 1 g/L, agitation speed 150 rpm and contact time of 100 min. Equations as well as the linear forms these isotherms are shown in Table 1 [6], [7], [8].

### 2.6 Adsorption kinetics

In the current study pseudo-first-order and pseudo-second-order kinetic models to determine the adsorption mechanism were investigated. The equations of these Kinetics are shown in Table 2 [9], [10], [11], [12].

### 2.7 Thermodynamic study

The thermodynamic study was carried out to determine the effect of temperature on the Cr(VI) adsorption. The thermodynamic parameters related to the adsorption process, such as the Gibbs free energy _(_ΔG∘,KJ/mol), entropy _(_ΔS∘,J/molK), and enthalpy _(_ΔH∘,KJ/mol_)_ changes were determined by using Vant Hoff according to Eqs. (10), (11), (12) [13]:

(10) ΔG∘=−RTLn(KL)

(11) Ln(KL)=(ΔS∘/R)−(ΔH∘/RT)

(12) ΔG∘=ΔH∘−TΔS∘

where K_L_ is the thermodynamic equilibrium constant (1/mol), R is the gas constant (8.314 J/mol k), T was the temperature (˚K), and ΔS∘,ΔH∘ were determined from the slope of linear regression between Ln K and 1/T according to Eqs. (10), (11).

## Figures and Tables

**Fig. S1:**